# Molecular Detection and Characterization of Coronaviruses in Migratory Ducks from Portugal Show the Circulation of *Gammacoronavirus* and *Deltacoronavirus*

**DOI:** 10.3390/ani12233283

**Published:** 2022-11-25

**Authors:** Mahima Hemnani, David Rodrigues, Nuno Santos, Sergio Santos-Silva, Maria Ester Figueiredo, Pedro Henriques, Joana Ferreira-e-Silva, Hugo Rebelo, Patricia Poeta, Gertrude Thompson, João R. Mesquita

**Affiliations:** 1 School of Medicine and Biomedical Sciences, Porto University, 4050-313 Porto, Portugal; 2 Coimbra College of Agriculture, Polytechnic of Coimbra, 3045-601 Coimbra, Portugal; 3 CEF, Forest Research Centre, Edifício Prof. Azevedo Gomes, ISA, Tapada da Ajuda, 1349-017 Lisboa, Portugal; 4 Centro de Investigação em Biodiversidade e Recursos Genéticos, InBIO Laboratório Associado, Universidade do Porto, 4485-661 Vairão, Portugal; 5 Microbiology and Antibiotic Resistance Team (MicroART), Department of Veterinary Sciences, Universidade de Trás-os-Montes e Alto Douro (UTAD), 5000-801 Vila Real, Portugal; 6 Veterinary and Animal Research Centre, Universidade de Trás-os-Montes e Alto Douro (UTAD), 5000-801 Vila Real, Portugal; 7 Associate Laboratory for Animal and Veterinary Science (AL4 Animals), Universidade de Trás-os-Montes e Alto Douro (UTAD), 5000-801 Vila Real, Portugal; 8 Epidemiology Research Unit (EPIUnit), Instituto de Saúde Pública da Universidade do Porto, 4050-600 Porto, Portugal

**Keywords:** coronavirus, migratory ducks, *Gammacoronaviruses*, *Deltacoronavirus*

## Abstract

**Simple Summary:**

Migratory birds have an enormous potential for dispersing pathogenic microorganisms. Ducks can host coronaviruses (CoVs), which have a high pathogenic expression and economic impacts, given their ability to migrate exceptional distances, facilitating the dispersal of microorganisms. This study aimed to identify and characterize the diversity of CoVs in migratory ducks from Portugal (*Anas platyrhynchos*, *Anas acuta,* and *Anas crecca*). Among the samples tested, 23 were characterized as *gammacoronavirus* and one as *deltacoronavirus.* The present study aimed to assess the circulation of CoVs in wild ducks from Portugal, being the first description of CoVs for these animals in Portugal.

**Abstract:**

Coronaviruses (CoVs) are part of the *Coronaviridae* family, and the genera Gamma (γ) and Delta (δ) are found mostly in birds. Migratory birds have an enormous potential for dispersing pathogenic microorganisms. Ducks (order Anseriformes) can host CoVs from birds, with pathogenic expression and high economic impact. This study aimed to identify and characterize the diversity of CoVs in migratory ducks from Portugal. Duck stool samples were collected using cloacal swabs from 72 individuals (*Anas platyrhynchos*, *Anas acuta,* and *Anas crecca*). Among the 72 samples tested, 24 showed amplicons of the expected size. Twenty-three were characterized as *Gammacoronavirus* and one as *Deltacoronavirus* (accession numbers ON368935-ON368954; ON721380-ON721383). The *Gammacoronaviruses* sequences showed greater similarities to those obtained in ducks (*Anas platyrhynchos*) from Finland and Poland, *Anas crecca* duck from the USA, and mute swans from Poland. Birds can occupy many habitats and therefore play diverse ecological roles in various ecosystems, especially given their ability to migrate exceptional distances, facilitating the dispersal of microorganisms with animal and/or human impact. There are a considerable number of studies that have detected CoVs in ducks, but none in Portugal. The present study assessed the circulation of CoVs in wild ducks from Portugal, being the first description of CoVs for these animals in Portugal.

## 1. Introduction

Coronaviruses (CoVs) belong to the *Coronaviridae* family, subfamily *Orthocoronavirinae*, having a positive-sense single-stranded RNA genome and an envelope equipped with protruding structures on their surface called spikes [1]. Its genome is one of the largest viral RNA genomes, with approximately 25–32 kb [2]. CoVs show a high genetic diversity that can be the result of their large genomes, high rates of mutation, infidelity of the RNA-dependent RNA polymerase, and high frequency of homologous RNA recombination [3].

The *Orthocoronavirinae* subfamily is divided into four genera based on genetic differences and serological cross-reactivity. *Alpha* and *Betacoronaviruses* might have a common ancestor, a CoV originating from bats [4]. For this reason, the viruses belonging to these two genera are found in bats [5] and other mammals, such as swine acute diarrhea syndrome coronavirus (SADS-CoV), transmissible gastroenteritis virus (TGEV), feline coronavirus (FCoV), bovine CoVs (BCoV), and rat coronavirus (RtCoV) [6,7]. On the other hand, *Gamma* and *Deltacoronavirus* evolved from a CoV originating in birds, with the majority of them causing diseases in birds, such as avian CoV infectious bronchitis virus (IBV), turkey CoV (TCoV), goose CoV, and duck CoV [8,9]. Both *Gamma* and *Deltacoronavirus* have been isolated and detected in wild and domestic birds in orders such as Anseriformes, Pelecaniformes, Ciconiiformes, Galliformes, Columbiformes. and Charadriformes [10,11,12]. However, *Gammacoronavirus* tends to be detected in domestic birds, while *Deltacoronavirus* infects both domestic and wild birds [13,14]. Two CoVs isolated from cetaceans have also been placed in the genus *Gammacoronavirus* [15,16], and *Deltacoronavirus* has also been found in pigs (porcine deltacoronavirus -PDCoV), causing acute diarrhea and dehydration [17]. Coronaviral infections have received significant attention from both the public and researchers [18].

CoVs have been identified in almost 15 orders of Aves, especially Charadriiformes (seagulls, plovers, sandpipers) and Anseriformes (ducks, geese, swans) [1]. Migratory birds have a huge potential for the transport and dispersal of a large number of pathogenic microorganisms [19]. Ducks, species from the Anseriformes order, can host a number of RNA viruses, including avian CoVs, and avian paramyxovirus type 1, with emerging evidence that suggests they may also be hosts of an array of avian astroviruses [20]. Infections by avian CoVs are characterized by acute, highly contagious, and economically important diseases in domesticated poultry [21]. However, the genetic diversity, evolution, distribution, and taxonomy of some CoVs dominant in birds still remain enigmatic [22]. Therefore, to add knowledge to this specific topic and to understand the role of circulation and the potential transboundary introduction of exotic avian CoVs, this study aimed to identify and characterize the diversity of CoVs in migratory ducks from Portugal.

## 2. Materials and Methods

### 2.1. Sample Collection

**Figure 1:**
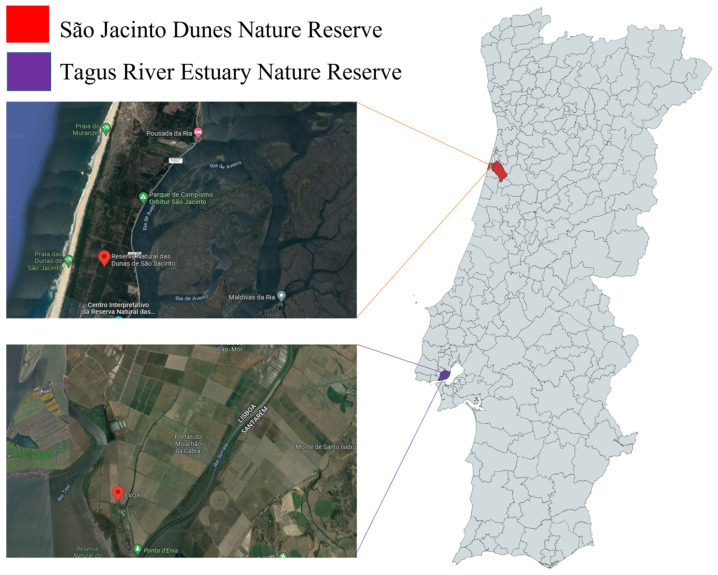
Selected sampling locations (city of Porto and Aveiro) in Portugal used in this study.

Samples of duck feces were collected on duck cloaca using cotton swabs and conserved at −23 ℃ from ducks captured for marking within duck ecology and migration studies. The species sampled were Mallard *Anas platyrhynchos*, Pintail *Anas acuta,* and Teal *Anas crecca*. Captures were performed in São Jacinto Dunes Nature Reserve (Aveiro) and at EVOA (in Tagus River Estuary Nature Reserve, Vila Franca de Xira) since these are areas of high concentration of ducks where long-term duck ecology studies have been performed (see Figure 1). Ducks were visually marked with nasal saddles and could be followed on the field. A license to capture and mark ducks was obtained from Instituto da Conservação da Natureza e das Florestas (ICNF), Portugal (permit number 40/2021).

Fecal swabs were thoroughly mixed by vortexing in 500 µL of phosphate-buffered saline (PBS) pH 7.2. RNA was extracted from the fecal suspension using the QIAamp viral mini kit (Qiagen, Hilden, Germany) according to the manufacturer’s instructions using 140 µL of the clarified supernatants. Eluted RNA was then kept at −80 °C until further processing. 

### 2.2. Screening for Coronaviruses 

Extracted nucleic acids were tested for CoVs using a broad-spectrum pan-CoV nested RT-PCR assay targeting the RNA-dependent RNA polymerase (RdRp)-conserved region with a final product size of 440 bp [23]. The sensitivity of the nested pan-CoV primers has been recently compared with different protocols by combining existing primers from different studies showing high performance and combining the chances of detecting known and unknown CoVs from all matrices [23].

We employed the one-step RT-PCR kit from GRiSP^®^, Porto, Portugal, for the initial round of PCR. The following conditions were used in the Veriti 96-well thermal cycler (Thermo Fisher) for amplification reactions with positive and negative controls: an initial cycle of 3 min at 95 °C, followed by 40 cycles of 95 °C for 15 s, 50 °C for 15 s, and 72 °C for 2 s, with a final elongation at 72 C for 10 min. Then, 2 μL of the first round’s products was utilized as a template for the second round using the Xpert Fast Hotstart Mastermix (2 x) with dye (GRiSP^®^, Porto, Portugal). A final amount of 25 μL was used for the PCR. The same thermal cycler was used to perform the amplification reactions with the positive and negative controls. The following conditions were used: an initial cycle of 3 min at 95 °C, 40 cycles of 95 °C for 15 s, 52 °C for 15 s, and 72 °C for 2 s, followed by an elongation at 72 °C for 10 min.

In order to identify the target DNA fragments, PCR amplification products were electrophoresed at 120 V for 30 min on a 1% agarose gel stained with Xpert Green Safe DNA gel stain (Grisp, Porto, Portugal). Molecular weights were assessed using a DNA weight comparison (100 bp DNA ladder; Grisp, Porto, Portugal).

### 2.3. Sanger Sequencing and Phylogenetic Analysis

Amplicons of the expected size were purified using the GRS PCR Purification Kit (Grisp, Porto, Portugal). Bidirectional sequencing was then performed using the target gene’s specific primers by the Sanger method. Sequence alignment was performed using the Bi-oEdit Sequence Alignment Editor v7.1.9 software package, version 2.1 (Ibis Biosciences, Carlsbad, CA, USA). The obtained sequences were trimmed, and consensus sequences were compared with the sequences found online in the nucleotide database NCBI (Gen-Bank, Carlsbad, CA, USA).

The viral sequences obtained in this study were submitted to GenBank under the accession numbers ON368935-ON368954 and ON721380-ON721383. These sequences, together with 36 reference strains from the 4 genera (*Alpha-*, *Beta-*, *Gamma-,* and *Deltacoronavirus*) obtained from GenBank, were aligned using MEGA 11 software [24]. Models function on MEGA 11 was used to opt for the model with the smallest Bayesian information criterion (BIC) score [25] using the maximum likelihood method, based on the general time reversible model using a discrete Gamma distribution and assuming evolutionarily invariable sites, 1000 bootstraps replicated, followed by editing with the Interactive Tree of Life (iTOL) platform [26].

## 3. Results

**Figure 2:**
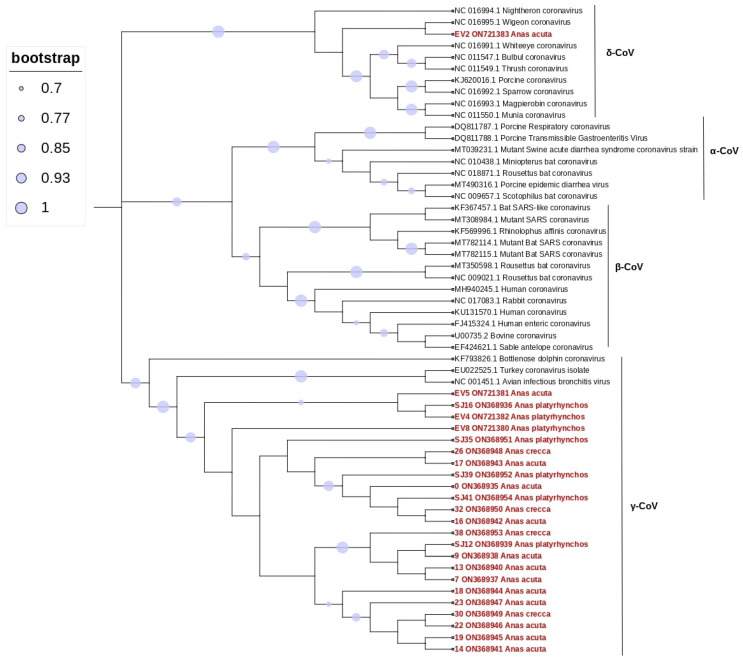
Phylogenetic tree constructed for the *alpha*, *beta*, *gamma*, and *delta* coronavirus, using 36 reference strains and 24 strains identified in this study. Phylogenetic analysis was based on a 406 nt partial region of the RdRp. The tree was constructed using MEGA 10 using the maximum likelihood based on the GTR + G model, and 1000 bootstraps were replicated. Samples from this study are indicated in red.

Among the total 72 samples tested, 24 presented amplicons of the expected size (33.3%; 95% confidence interval [CI]: 22.7–45.4). Bidirectional sequencing of these 24 products, followed by nucleotide BLAST analysis, showed that the majority (n = 23) were characterized as *Gammacoronavirus* (31.4%; 95% CI: 21.4–44.0)*,* and one was characterized as *Deltacoronavirus* (1.4%; 95% CI: 0.03–7.5), as shown in Figure 2. Pintail showed a higher prevalence, with 12 of 24 samples positive (48%). Mallard, a resident species in Portugal (Rodrigues et al. 2000), also had positive samples.

Sequence analysis within the obtained CoV sequences showed identities ranging from 94% and 100%. Characterization by BLAST indicated that the sequences showed the highest hits (97.76–99.44%) to those sequences obtained from ducks from Finland, Poland, and the USA and mute swans from Poland. Phylogenetic analysis using the obtained 24 CoV sequences and 36 reference strains confirmed the classification as *Gammacoronaviruses* and *Deltacoronavirus* (Figure 2).

The obtained phylogenetic tree showed that the retrieved sequences in this study clustered together with those obtained from a bottlenose dolphin (bottlenose dolphin coronavirus), from a chicken (avian infectious bronchitis virus), and a turkey (turkey coronavirus), with all of them clustering in *Gammacoronavirus*, except for one pintail sample, which clustered with a Wigeon *Anas penelope* (Wigeon coronavirus HKU20) *Deltacoronavirus*. Details of the samples from this study can be found in Table 1.

## 4. Discussion

The present study assessed the circulation of CoVs in wild ducks from Portugal, being the first description of CoVs for these animals in Portugal. We have also shown that the CoV strains found in the duck population under study are closely related to the *gammacoronavirus* strains retrieved from duck species *Anas platyrhynchos* from Poland and Finland [1,2,27], mute swans from Poland [1], the duck species *Anas crecca* from Hong Kong [11] and the USA (accession number: KJ741882).

As it is known, the *Alphacoronavirus* and *Betacoronavirus* infect mostly mammals, while *gammacoronavirus* and *deltacoronavirus* mainly infect birds [8,9]. While there is a growing number of studies focusing on the presence of coronavirus in bats, there is a variety of CoVs known to circulate among wild and domestic birds as well [2], which, upon entering poultry production premises, may cause severe morbidity and mortality to the birds [28], causing substantial economic losses [29].

Several viruses, including zoonotic and economically significant pathogens, are known to circulate among wild birds [2,30]. The main representative of avian coronavirus is the infectious bronchitis virus (IBV) [31], which is a *Gammacoronavirus*, a highly contagious viral disease that is considered responsible for significant economic losses in the poultry industry worldwide (Moreno et al., 2017). IBV control has been hampered by the intricate IBV evolution over the years, by the emergence of many different antigenic or genotypic types, commonly referred to as variants, that are likely to be facilitated by the spillover through migratory birds [32]; hence, continued monitoring of CoVs in migratory birds is key to mitigating potential outbreaks.

Birds can occupy many habitats and therefore serve diverse ecological roles in various ecosystems [33], especially because of their ability to migrate exceptional distances [34]. The most frequently proposed reason for birds’ migration is to benefit from a seasonal availability of resources, to breed, and also to avoid harsh winters [35]. This ability of dispersion can also, unfortunately, facilitate the dispersion of microorganisms with animal and/or human impact [2]. Hence alerts should be made towards surveillance of potentially pathogenic viruses in wild birds.

*Gammacoronavirus* and *Deltacoronavirus* were detected in a large variety of birds from different countries, such as Sweden [36], Norway [37], England [38], South Korea [39], and Australia [40] in either aquatic or nonaquatic variety, which highlights the potential for viral spillover to domestic species. There is a considerable number of studies that detected CoVs in ducks, but none in Portugal. Ducks are able to occupy diverse ecological niches and are either migratory or resident [1]. The strains of CoV found in our study are closely related to the strains from Finland, and Finnish mallards are strongly migratory [41]. Some species have adapted to urbanized landscapes, increasing their chances of being in contact with humans. The potential for transmissibility between bird species, even between wild and captive members of the same species (e.g., *Anas platyrhynchos*), or between other species, could trigger a captive poultry outbreak, especially in extensively farmed birds, leading to large economic losses in a wide geographical area [42].

Wildlife has been under epidemiological surveillance to identify its possible roles as a reservoir for emerging viruses that may pose a risk to humans and threaten wildlife [21]. Our investigation showed the presence of *Gammacoronavirus* and *Deltacoronavirus* in ducks, indicating that they harbor CoVs and possibly spread it. It is important to continue surveillance of wild ducks for the presence of avian coronavirus to understand better population flyways and ducks’ migration, especially the ones with close contact with humans, to understand better the evolution and ecology of coronaviruses, and to monitor emerging new strains that can possibly cause more losses to the poultry industry and transmit to humans [43]. The use of nasal saddles on sampled ducks can also allow the study of possible impacts of CoVs on wild duck species’ survival.

## 5. Conclusions

This study identified *Gammacoronavirus* and *Deltacoronaviruses* in migratory ducks from Portugal, and it is the first report of CoVs for these animals in the country. Migratory birds can occupy many habitats, primarily because of their ability to migrate exceptional distances. This ability of dispersion can also, unfortunately, facilitate the dispersion of microorganisms with animal and/or human impact. Hence, efforts should be made towards the surveillance of potentially pathogenic viruses in wild birds and for monitoring emerging new strains that can possibly cause more losses to the poultry industry and transmit to humans.

## Figures and Tables

**Table 1:** Details of the samples from this study.

Collection Site	Sample ID	Host Species	Accession Number	CoV Genera
*Evoa*	#0	*Anas acuta*	ON368935	*Gammacoronavirus*
	#7	*Anas acuta*	ON368937	*Gammacoronavirus*
	#9	*Anas acuta*	ON368938	*Gammacoronavirus*
	#13	*Anas acuta*	ON368940	*Gammacoronavirus*
	#14	*Anas acuta*	ON368941	*Gammacoronavirus*
	#16	*Anas acuta*	ON368942	*Gammacoronavirus*
	#17	*Anas acuta*	ON368943	*Gammacoronavirus*
	#18	*Anas acuta*	ON368944	*Gammacoronavirus*
	#19	*Anas acuta*	ON368945	*Gammacoronavirus*
	#22	*Anas acuta*	ON368946	*Gammacoronavirus*
	#23	*Anas acuta*	ON368947	*Gammacoronavirus*
	#26	*Anas crecca*	ON368948	*Gammacoronavirus*
	#30	*Anas crecca*	ON368949	*Gammacoronavirus*
	#32	*Anas crecca*	ON368950	*Gammacoronavirus*
	#38	*Anas crecca*	ON368953	*Gammacoronavirus*
	#EV2	*Anas acuta*	ON721383	*Deltacoronavirus*
	#EV4	*Anas platyrhynchos*	ON721382	*Gammacoronavirus*
	#EV5	*Anas acuta*	ON721381	*Gammacoronavirus*
*S São Jacinto Dunes Nature Reserve*	##EV8	*Anas platyrhynchos*	ON721380	*Gammacoronavirus*
	#SJ12	*Anas platyrhynchos*	ON368939	*Gammacoronavirus*
	#SJ16	*Anas platyrhynchos*	ON368936	*Gammacoronavirus*
	#SJ35	*Anas platyrhynchos*	ON368951	*Gammacoronavirus*
	#SJ39	*Anas platyrhynchos*	ON368952	*Gammacoronavirus*
	#SJ41	*Anas platyrhynchos*	ON368954	*Gammacoronavirus*

## Data Availability

The data presented in this study are available on request from the corresponding author.
